# The response of porcine monocyte derived macrophages and dendritic cells to *Salmonella* Typhimurium and lipopolysaccharide

**DOI:** 10.1186/s12917-014-0244-1

**Published:** 2014-10-01

**Authors:** Kamila Kyrova, Hana Stepanova, Ivan Rychlik, Ondrej Polansky, Lenka Leva, Zuzana Sekelova, Martin Faldyna, Jiri Volf

**Affiliations:** Veterinary Research Institute, Hudcova 70, Brno, 621 00 Czech Republic

**Keywords:** Macrophage, Dendritic cell, Porcine, LPS, *Salmonella*, Response

## Abstract

**Background:**

Following infection and initial multiplication in the gut lumen, *Salmonella* Typhimurium crosses the intestinal epithelial barrier and comes into contact with cells of the host immune system. Mononuclear phagocytes which comprise macrophages and dendritic cells (DC) are of key importance for the outcome of *Salmonella* infection. Although macrophages and DC may differentiate from a common precursor, their capacities to process and present antigen differ significantly. In this study, we therefore compared the response of porcine macrophages and DC differentiated from peripheral blood monocytes to *S*. Typhimurium and one of the most potent bacterial pathogen associated molecular patterns, bacterial lipopolysaccharide. To avoid any bias, the expression was determined by protein LC-MS/MS and verified at the level of transcription by quantitative RT-PCR.

**Results:**

Within 4 days of culture, peripheral blood monocytes differentiated into two populations with distinct morphology and expression of MHC II. Mass spectrometry identified 446 proteins in macrophages and 672 in DC. Out of these, 433 proteins were inducible in macrophages either after infection with *S*. Typhimurium or LPS exposure and 144 proteins were inducible in DC. The expression of the 46 most inducible proteins was verified at the level of transcription and the differential expression was confirmed in 22 of them. Out of these, 16 genes were induced in both cell types, 3 genes (VCAM1, HMOX1 and Serglycin) were significantly induced in macrophages only and OLDLR1 and CDC42 were induced exclusively in DC. Thirteen out of 22 up-regulated genes contained the NF-kappaB binding site in their promoters and could be considered as either part of the NF-kappaB feedback loop (IkappaBalpha and ISG15) or as NF-kappaB targets (IL1beta, IL1alpha, AMCF2, IL8, SOD2, CD14, CD48, OPN, OLDLR1, HMOX1 and VCAM1).

**Conclusions:**

The difference in the response of monocyte derived macrophages and DC was quantitative rather than qualitative. Despite the similarity of the responses, compared to DC, the macrophages responded in a more pro-inflammatory fashion.

**Electronic supplementary material:**

The online version of this article (doi:10.1186/s12917-014-0244-1) contains supplementary material, which is available to authorized users.

## Background

Pigs are one of the most important sources of animal proteins for the human population. However, due to intensive rearing, pigs are also highly susceptible to various pathogens including those with zoonotic potential. *Salmonella enterica* serovar Typhimurium is an example of a zoonotic agent for which pigs represent a reservoir for the human population. In fact, pigs represent the second most common source of *Salmonella* for humans after poultry and are the most important source if only serovar *S*. Typhimurium is considered.

*Salmonella* in pigs is transmitted by the oral-fecal route. Infections of pigs with *S*. Typhimurium are often asymptomatic [1] although mild diarrhea may be recorded at the early stage of infection [2,3]. After initial multiplication in the gut lumen, *S*. Typhimurium invades the intestinal epithelial cells and comes into contact with the host’s immune system [4]. The innate immune responses of different parts of porcine intestinal tract have been repeatedly described [5–7]. Of the different leukocyte subpopulations, mononuclear phagocyte cells are of key importance for the outcome of *Salmonella* infection which comprise of macrophages (MΦ) and dendritic cells (DC). When these cells come into contact with bacterial pathogens, they recognize the pathogens through the presence of pathogen associated molecular patterns (PAMP) present in prokaryotic pathogens but absent from eukaryotic host cells. This leads to the modification of gene expression in MΦ and DC, and secretion of signaling molecules to coordinate responses of other cells of the host immune system. In addition, both MΦ and DC are able to take up, process and present antigens to lymphocytes, thereby inducing the development of an adaptive immune response [8,9].

Although only particular subsets of MΦ and DC can be differentiated from a monocyte precursor, their capacity to take up, process and present antigen differ significantly. There are several papers to date describing the expression profiles of selected genes in porcine monocytes, dendritic cells or macrophages in response to external stimuli [10–15]. However, these studies compared the responses either monocytes and monocyte derived dendritic cells (MoDC) [13], two differently generated macrophages [15] or two different DC populations [11,12]. The response of MΦ and DC derived from monocytes has never been compared. Moreover, all the studies either determined the expression of preselected genes such as TLRs, MHC-II molecules, chemokines and cytokines by quantitative RT-PCR or used the Affymetrix microarray, so the measurements were limited to the level of transcription. Since the general understanding of antigen presentation and associated processes, especially in a porcine model, is far from being completely understood, in this study, we therefore differentiated porcine monocyte-derived macrophages (MoMΦ) and MoDC, and compared their response to *S*. Typhimurium and one of the most potent bacterial PAMP, bacterial LPS. To avoid any bias, expression was determined by protein LC-MS/MS and verified at the level of transcription by QRT-PCR. Using such an approach, we concluded that the difference in the response of MoMΦ and MoDC was quantitative rather than qualitative, i.e. MoDC responded less extensively than MoMΦ to LPS or *S.* Typhimurium stimulation.

## Results

### Cell differentiation

**Figure 1:**
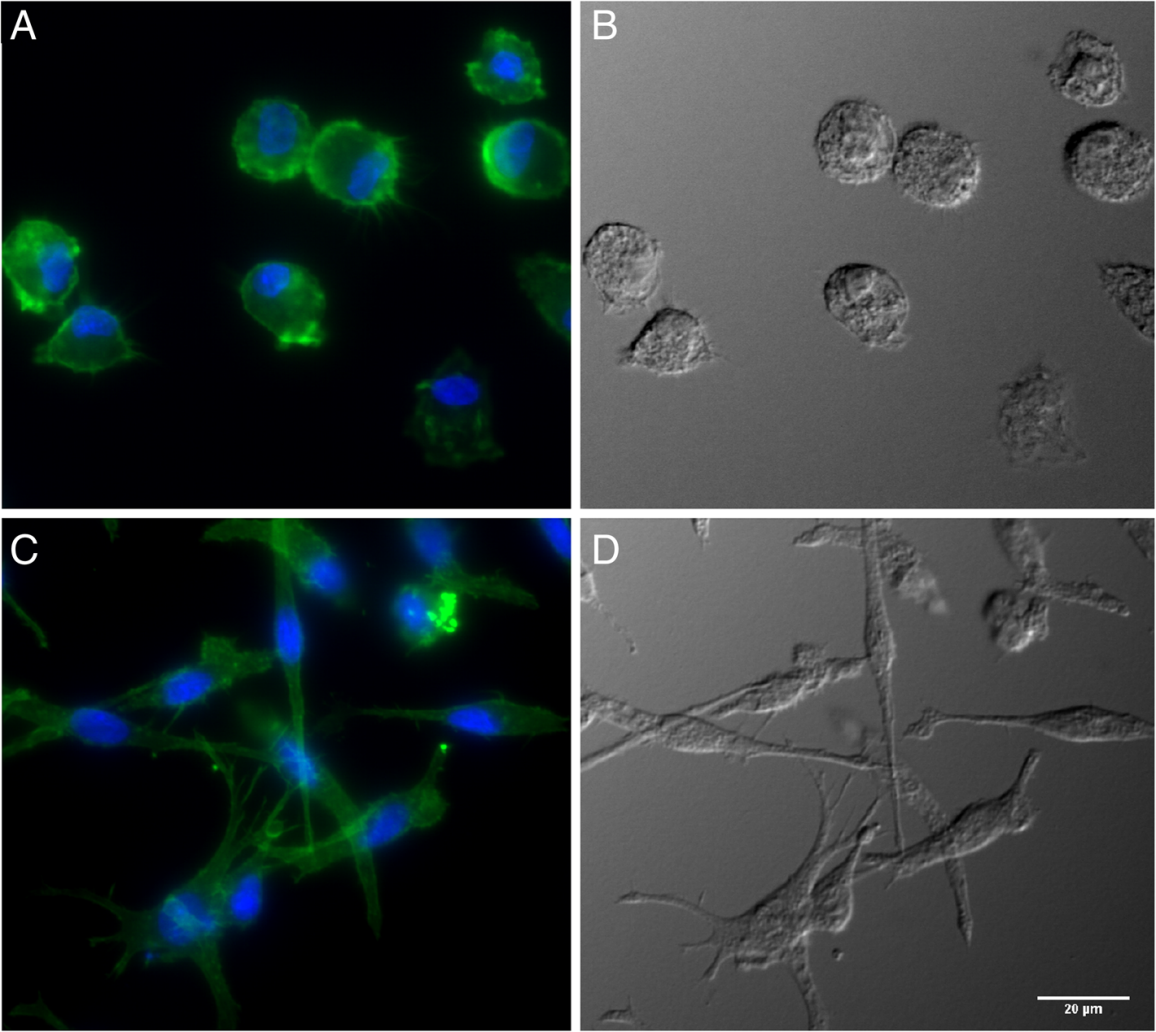
**Cell morphology after differentiation of peripheral blood monocytes.** Fluorescence microscopy and DIC was used to visualize differences in cell morphology. **A** and **B**, MoMΦ, **C** and **D**, MoDC. **A** and **C**, fluorescence microscopy with nuclei stained in blue with DAPI and cytoskeleton stained in green with phalloidin. **B** and **D**, DIC.

Depending on culture conditions, peripheral blood monocytes differentiated into two populations with distinct morphology within 4 days of culture. Adhered monocytes differentiated into MoMΦ of spherical shape with characteristic short hairy protrusions on their surface. On the other hand, monocytes treated with IL4 and GM-CSF differentiated into MoDC characteristic by elongated cells with numerous dendrites typical of dendritic cells (Figure 1).

**Figure 2:**
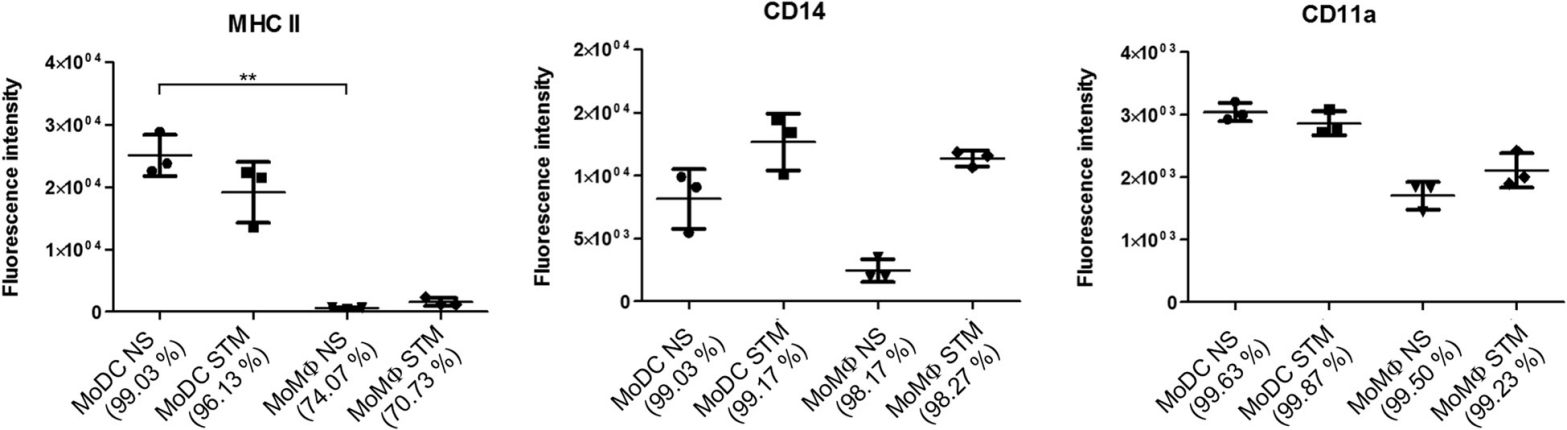
**Surface markers of MoMΦ and MoDC differentiated from PBMC of three donor pigs.** The Y axis represents the mean fluorescence intensity measured for a particular surface molecule with the error bar representing SD. The percentages of positive cells for individual marker are mentioned in parenthesis and were calculated as a mean of three experiments.

Flow cytometric analysis further confirmed the difference between the two cell populations. The most remarkable difference was a more than 40 times higher expression of MHC-II molecules on the surface of MoDC compared to MoMΦ. The expression of CD14 and CD11a, when compared to MoMΦ, was numerically but not significantly higher in MoDC (Figure 2). On the other hand, expression of CD172α, CD16, CD163, CD45, TLR-2 and TLR-4 did not differ between both cell types (data not shown). The expression of surface molecules on MoDC and MoMΦ was also determined after *S*. Typhimurium infection. In response to infection, the amount of CD14 increased in both cell types whereas the expression of the remaining cell surface molecules did not change (Figure 2).

### Protein mass spectrometry

The previous experiment showed that CD14 increased both in MoMΦ and MoDC after *S*. Typhimurium infection. In the next experiment, we therefore determined what other proteins might have increased in expression in both MoMΦ and MoDC. In addition to *S*. Typhimurium infection, protein expression was also determined in the LPS exposed cell cultures.

**Figure 3:**
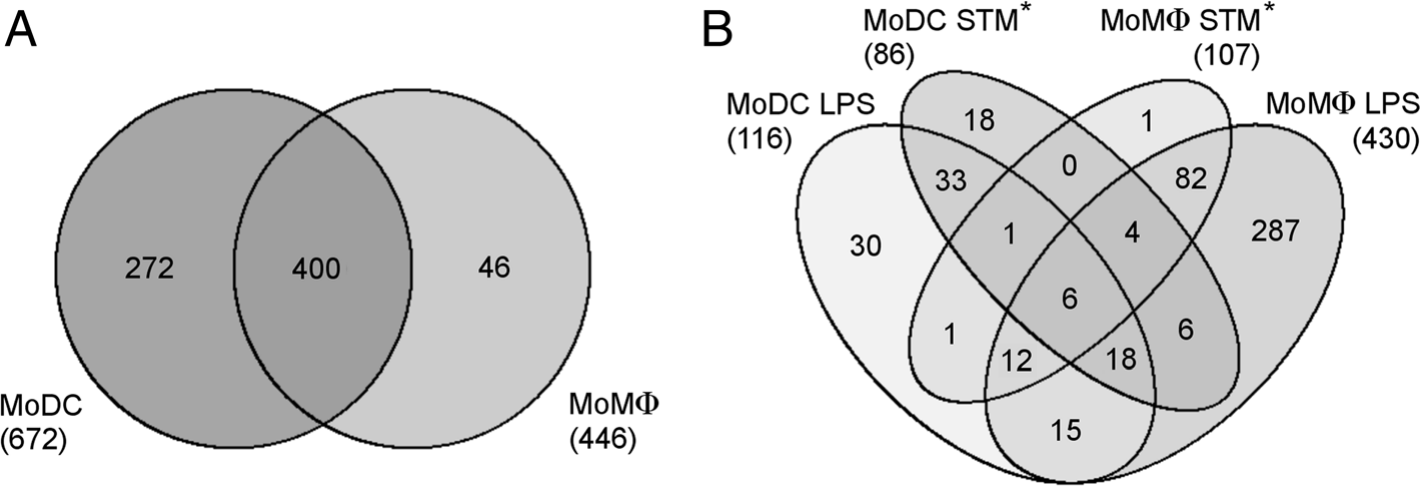
**Numbers of proteins identified in MoMΦ and MoDC by LC-MS/MS analysis.** Panel **A** Proteins expressed in both or either of the cell types. The number of proteins specific for each cell type might be actually lower as these were calculated after applying all filtering criteria described in Methods. Some of the MoDC proteins considered as specific in this figure can be low level expressed also in macrophages. The higher number of proteins expression of which was found to be induced twofold or more following individual stimulations (panel **B**) shows higher reactivity of MoMΦ in comparison with MoDC. *STM - *S*. Typhimurium.

Mass spectrometry detected at least once 2191 proteins in MoMΦ and 2204 proteins in MoDC [see Additional file 1]. However, when we applied the filter criteria described below, the number of proteins decreased to 446 proteins which were repeatedly detected in MoMΦ and 672 in MoDC (Figure 3A). Out of the filtered proteins, 400 were detected in both MoMΦ and MoDC, 46 proteins were identified only in MoMΦ and 272 proteins were identified only in MoDC. The comparison of the MoMΦ and MoDC response to LPS and *S*. Typhimurium showed that MoMΦ were more responsive as the number of proteins induced more than 2 fold was higher in MoMΦ than in MoDC (Figure 3B). The MoMΦ also showed a higher mean fold induction than the MoDC when comparing the proteins induced in both cell types [see Additional file 1].

### QRT-PCR

Since mass spectrometry was performed in cells originating from only two donor pigs (Table 1), proteomic expression data were therefore confirmed at the transcriptional level by quantitative real-time PCR to which additional samples from 3 different pigs were included. Genes selected for QRT-PCR verification included those in which the summed fold inductions determined by LC-MS/MS following LPS stimulation and *S*. Typhimurium infection exceeded a factor of 20 in the case of the less responsive MoDC, or a factor of 30 in the case of the more responsive MoMΦ. The selection resulted in 46 genes/proteins (see Additional file 2) and the differential expression was confirmed by QRT-PCR in 22 genes (Table 2).

**Table 1:** **List of samples used in this study**

**Pig**	**Cell culture**	**LC MS (NS/LPS/STM** ^**†**^ **)**	**QRT PCR**	**FACS**	**Light microscopy**
1	A			Full FACS analysis*	Yes
2	A			Full FACS analysis	Yes
3	A			Full FACS analysis	Yes
4	A^#^	Yes	Yes	CD14 check	Yes
	B^#^	Yes	Yes	CD14 check	Yes
5	A^#^	Yes	Yes	CD14 check	Yes
	B^#^	Yes	Yes	CD14 check	Yes
6	A		Yes	CD14 check	Yes
7	A		Yes	CD14 check	Yes
8	A		Yes	CD14 check	Yes

^†^ *NS* non stimulated cells, *STM S*. Typhimurium.

*Full analysis comprises the FACS measurement of the following surface markers: CD11a, CD14, CD16, CD45, CD163, CD172α, MHC-II, TLR2, TLR4. FACS analysis was performed only for untreated and *S.* Typhimurium infected cell cultures.

^#^Two independent batches of MoMΦ and MoDC were obtained from these pigs.

**Table 2:** **Fold inductions of genes identified in this study in MoMΦ or MoDC in response to LPS or** ***Salmonella*** **Typhimurium (STM) exposure determined by QRT-PCR**

	**MoDC**	**MoDC fold induction** ^**$**^		**MoMΦ**	**MoMΦ fold induction**		**MoMΦ to MoDC**		
	**Basal exp**	**STM**	**LPS**	**Basal exp**	**STM**	**LPS**	**NS**	**STM**	**LPS**
IL1B	0.06 ± 0.04	**106** ^**#**^ ± 101	**2358** ^**#**^ ± 1268	0.11 ± 0.06	**383** ^**#**^ ± 451	**1999** ^**#**^ ± 1743	n	*	n
AMCF2	0.40 ± 0.20	**35.3** ^**#**^ ± 33.0	**543** ^**#**^ ± 528	0.75 ± 0.52	**115** ^**#**^ ± 107	**305** ^**#**^ ± 267	n	n	n
CXCL2	0.71 ± 0.28	**17.4** ^**#**^ ± 19.1	**200** ^**#**^ ± 161	0.19 ± 0.19	**155** ^**#**^ ± 102	**668** ^**#**^ ± 432	n	n	n
IL1A	0.28 ± 0.14	**22.9** ^**#**^ ± 25.3	**229** ^**#**^ ± 164	0.24 ± 0.12	**86.5** ^**#**^ ± 48.8	**187** ^**#**^ ± 57	n	**	n
IL8	15.3 ± 13.7	5.9 ± 5.74	**34.2** ^**#**^ ± 21.6	6.78 ± 5.95	**22.9** ^**#**^ ± 13.2	**113** ^**#**^ ± 66	n	n	n
ISG15	0.88 ± 0.52	**2.94** ^**#**^ ± 1.22	2.00 ± 0.94	0.17 ± 0.10	**22.1** ^**#**^ ± 2.0	**78.9** ^**#**^ ± 104.4	***	n	n
MARCKSL1	0.70 ± 0.60	1.82 ± 0.63	**4.07** ^**#**^ ± 2.30	0.25 ± 0.08	**17.9** ^**#**^ ± 9.1	**42.9** ^**#**^ ± 15.6	n	*	**
SOD2	4.28 ± 4.58	2.13 ± 0.80	**12.8** ^**#**^ ± 12.9	4.61 ± 2.55	**11.3** ^**#**^ ± 4.6	**27.6** ^**#**^ ± 11.6	n	**	n
IκBα	1.15 ± 0.36	**2.74** ^**#**^ ± 1.12	**8.12** ^**#**^ ± 3.10	1.13 ± 0.57	**9.64** ^**#**^ ± 3.72	**15.1** ^**#**^ ± 4.8	n	***	n
CD14	0.52 ± 0.19	**3.84** ^**#**^ ± 1.52	**14.4** ^**#**^ ± 9.6	0.35 ± 0.27	**14.0** ^**#**^ ± 9.7	**18.0** ^**#**^ ± 20.0	n	n	n
PPA1	1.34 ± 0.84	**1.85** ^**#**^ ± 0.7	**4.83** ^**#**^ ± 1.93	1.39 ± 0.17	**4.42** ^**#**^ ± 1.00	**7.02** ^**#**^ ± 1.73	n	***	n
CD48	7.35 ± 4.25	1.83 ± 0.22	**3.2** ^**#**^ ± 1.8	9.93 ± 2.43	**3.34** ^**#**^ ± 0.71	**5.33** ^**#**^ ± 1.90	n	*	*
NF-κB1	1.57 ± 0.41	1.82 ± 0.61	**3.49** ^**#**^ ± 1.4	1.97 ± 0.44	**2.25** ^**#**^ ± 0.7	**4.63** ^**#**^ ± 2.20	n	n	n
ANXA1	15.3 ± 4.7	1.78 ± 0.9	**3.74** ^**#**^ ± 1.91	22.9 ± 13.4	1.40 ± 0.38	**2.45** ^**#**^ ± 1.01	n	n	n
OPN	86.1 ± 42.8	2.33 ± 1.5	**3.42** ^**#**^ ± 2.27	138 ± 72	2.12 ± 1.20	**3.81** ^**#**^ ± 2.00	n	n	n
PFDN2	0.09 ± 0.03	1.54 ± 0.51	**2.22** ^**#**^ ± 0.63	0.12 ± 0.04	**2.12** ^**#**^ ± 0.60	**2.63** ^**#**^ ± 0.72	n	n	n
PSMB4	6.18 ± 1.58	1.42 ± 0.30	**1.82** ^**#**^ ± 0.31	7.34 ± 1.39	1.73 ± 0.40	**2.11** ^**#**^ ± 0.54	n	n	n
**MoDC only**									
OLDLR1	9.73 ± 3.74	**1.95** ^**#**^ ± 0.5	**3.33** ^**#**^ ± 0.91	26.2 ± 3.0	1.43 ± 0.42	1.62 ± 0.41	***	*	n
CDC42	3.52 ± 0.70	**1.62** ^**#**^ ± 0.33	1.63 ± 0.33	4.78 ± 1.43	1.60 ± 0.13	1.53 ± 0.61	n	n	n
**MoMΦ only**									
VCAM1	0.33 ± 0.25	2.32 ± 1.90	5.52 ± 4.51	0.18 ± 0.19	**9.82** ^**#**^ ± 6.24	**18.9** ^**#**^ ± 13.6	n	n	n
HMOX1	0.32 ± 0.20	2.32 ± 1.53	1.42 ± 0.51	0.45 ± 0.11	**2.34** ^**#**^ ± 1.11	**2.44** ^**#**^ ± 0.14	n	n	*
Serglycin	45.9 ± 19.9	1.52 ± 0.71	2.40 ± 1.03	35.7 ± 28.9	**3.04** ^**#**^ ± 1.32	**2.84** ^**#**^ ± 1.33	n	n	n

^$^The fold inductions are presented as mean ± SD calculated based on ratios of individual treated samples relative to the mean of appropriate non-stimulated control.

^#^The numbers signed with ^**#**^represent significantly different results compared to non-stimulated (NS) cells.

*Statistically significant differences between MoMΦ and MoDC with the same treatment. *p < 0.05; **p < 0.01; ***p < 0.001; n = non-significant.

Out of these, 16 genes were induced both in MoMΦ and MoDC, 3 genes (VCAM1, HMOX1 and Serglycin) were significantly induced in MoMΦ only and OLDLR1 and CDC42 were significantly induced exclusively in MoDC. Out of the 16 genes up-regulated in both cell types, 5 the most up-regulated ones encoded cytokines (IL1β, AMCF2 (the porcine homolog of CXCL 5/6), CXCL2, IL8 and IL1α). In agreement with the proteomic data, the fold inductions determined by QRT-PCR were usually higher in MoMΦ than in MoDCs, despite the fact that the differences between MoMΦ and MoDC responsiveness reached statistical significance only in 2 genes after exposure to LPS and 7 genes after infection with *S*. Typhimurium. This also means that the responsiveness of MoMΦ and MoDCs to LPS was similar but DCs, unlike MΦ, did not extensively respond to *S*. Typhimurium infection.

## Discussion

In this study we were interested in the interactions of porcine MoMΦ and MoDCs with LPS and *S*. Typhimurium. Both cell morphology and flow cytometry indicated that the culture conditions led to the differentiation of distinct cell types. Expression of surface molecules observed on MoDC was in agreement with the previous observations [10,16]. The up-regulation of CD14 in porcine alveolar macrophages following stimulation with LPS or *Salmonella* infection was also reported earlier [10,17].

Similar responses to free LPS and *S*. Typhimurium of both cell types indicate that LPS is one of the dominant antigens of *S*. Typhimurium. The infection of MoMΦ with *S*. Typhimurium did cause induction of only 3 additional proteins compared to LPS stimulated cells (Figure 3B). In case of MoDC there were 28 proteins solely induced by *S*. Typhimurium which represents less than one fifth of all proteins induced in MoDC. The higher stimulating potential of LPS compared to infection with *S*. Typhimurium was likely caused by the soluble character of LPS which was present in media and homogenously stimulated all the cells. On the other hand, only about 10% of cells were invaded by *S*. Typhimurium (data not shown), so the remaining uninfected cells could respond only to LPS released from bacterial cells into the medium during the experiments. The effect of indirect stimulation of infected cells toward their uninfected by-standers remains to be determined. Both LC-MS/MS and real-time PCR showed that the differences in the expression of genes inducible both in MoMΦ and in MoDC were more pronounced in MoMΦ. Together with the high expression of MHCII, this shows that the primary role of DC is antigen presentation whereas macrophages are involved in modulation of the environment by cytokine signaling.

Eleven genes/proteins, IL1β, AMCF2, CXCL2, IL8, IL1α, ISG15, MARCKSL1, SOD2, IκBα, CD14 and VCAM were induced by more than 10 fold. Out of these, IL1β, CXCL2, AMCF2, IL8, IL1α and the secreted form of ISG15 have a cytokine and/or chemokine function and their expression is related to the NF-κB dependent proinflammatory pathway. The central role of the NF-κB pathway is further supported by the fact that more than half of the identified genes (13 of 22) contain the NF-κB binding site in the promoter. These genes could be considered as either involved in the NF-κB feedback loop (IκBα and ISG15) [14,18] or act as NF-κB targets: IL1β, IL1α, AMCF2, IL8, SOD2, CD14, CD48, OPN, OLDLR1, HMOX1 and VCAM1 [17–28]. IL1β, AMCF2, IL8, CXCL2, IL1α, MARCKSL1, SOD2, PPA1 and ISG15 were also found among the top 10% of the most inducible genes of bone marrow derived porcine macrophages treated with LPS [15].

The differences between MoMΦ and MoDC responses were quite low. This could be caused by the fact that we selected the most inducible and highly abundant proteins, which had similar expression profiles following exposure to LPS or *S*. Typhimurium, and minority proteins responsible for the specificity of MoMΦ and MoDC remained unrecognized. The exceptions were represented by MARCKS-related gene/protein (MARCKSL1). This gene was expressed and induced in MoMΦ after exposure to both LPS and *S*. Typhimurium at a significantly higher rate than in MoDC. Finally, its induction in LPS stimulated MoMΦ was more than 40 fold indicating that this was a highly inducible gene.

MARCKSL1 (synonyms MLP, MacMARCKS, F52) is expressed mainly in the brain, reproductive tissues, and macrophages [29,30] and belongs to a family of the unstructured proteins that mediate cross-talk in cell-to-cell signaling. MARCKSL1 is involved in the regulation of cell migration, adhesion and phagocytosis, as well as in neurosecretion and brain development [31–33]. Transcription of MARCKSL1 is also strongly increased upon stimulation with bacterial LPS [34–37]. Mancek-Keber et al. [38] showed that the MARCKSL1 protein binds LPS with an affinity sufficient enough to sequester a large fraction of the internalized LPS or intracellular Gram-negative bacteria within the endosome/phagosome.

## Conclusions

In this study we have shown that *S*. Typhimurium and LPS in particular represent one of the most potent activators of signaling pathways of MoMΦ and MoDC leading to a similar response in both cell types. Despite the similarity of the responses, compared to MoDC, the MoMΦ were more pro-inflammatory. This difference was less obvious in response to LPS and more obvious in response to *S*. Typhimurium. Our results showed that increased pro-inflammatory signaling by MoMΦ compared to MoDC might be, at least in some subsets of mononuclear phagocytes, associated with differential MARCKSL1 protein expression though the exact role of these proteins in differential responsiveness of MoMΦ and MoDC to *S*. Typhimurium infection remains to be determined.

## Methods

### Differentiation of monocyte-derived macrophages and dendritic cells

Blood was collected from healthy 8-10 month old conventional pigs kept at the Veterinary Research Institute for the educational purposes. The animal care protocol for this experiment followed the Czech guidelines for animal experimentation and was approved by the Branch Commission for Animal Welfare of the Ministry of Agriculture of the Czech Republic (permission No MZe 921). Peripheral Blood Mononuclear cells (PBMC) were isolated by gradient centrifugation using Histopaque-1077 (Sigma-Aldrich). Monocytes were sorted from PBMC using indirect magnetic labelling based on the expression of CD14. In the first step, anti-CD14 monoclonal antibody (MIL-2, IgG2b, AbD Serotec) was used and consequently, the CD14-positive cells were captured by goat anti-mouse IgG MicroBeads (Miltenyi Biotec) and sorted using a QuadroMACS separator (Miltenyi Biotec) according to manufacturer’s recommendations. The purity of the sorted cells was checked using a flow cytometer (LSR Fortessa, Becton Dickinson) with more than 90% of the cells being CD14 positive. Approx. 5 × 10^5^ CD-14 positive monocytes resuspended in 1 ml of media were seeded per well on a 24-well microplate.

Half of the isolated monocytes were differentiated using protocol described previously [39] and the resulting cells were considered as MoDC throughout the study. RPMI-1640 medium (PAA) supplemented with antibiotics penicillin/streptomycin (Sigma-Aldrich), 10% heat-inactivated fetal calf serum (PAA), recombinant porcine IL4 (50 ng/ml, R&D Systems) and GM-CSF (20 ng/ml, R&D Systems) was used. The second half of the cells were left to adhere to plastic microplates in DMEM (Gibco Invitrogen) supplemented with penicillin/streptomycin (Sigma-Aldrich) and 10% heat-inactivated and filtered porcine serum (PAA) as described previously [40]. These cells were considered as MoMΦ for the purposes of this study. Both MoMΦ and MoDC were differentiated at 37°C in a 5% CO_2_ incubator for 4 days. MoMΦ and MoDC were differentiated from peripheral blood monocytes from 8 different pigs, although not all of the cultures were used for all of the analyses (Table 1).

### Fluorescence microscopy and flow cytometry

Differentiation of MoMΦ and MoDC was confirmed by flow cytometry and light microscopy. For light microscopy, the cells were grown on 13 mm glass slides as described above. After a 4-day culture, the cells were fixed with 4% paraformaldehyde and labelled with DAPI as a nuclear stain and Alexa Fluor (AF) 488 conjugated phalloidin (Invitrogen) to visualize the actin cytoskeleton. Microscopy was performed using epifluorescence inverted microscope Olympus IX81 equipped with PlanSAPO 40× (NA 0.95) objective using differential interference contrast (DIC), and fluorescence mode to detect DAPI and AF-488 fluorescence.

Cells for flow cytometry were harvested by 0.2% EDTA, washed in PBS and labelled with the following antibodies against surface proteins: anti-CD11a (BL1H8, IgG2b, AbD Serotec), anti-CD14 (MIL-2, IgG2b, AbD Serotec), anti-CD16 (G7, IgG1, AbD Serotec), anti-CD45 (K252.1E4, IgG1, gift from Dr. K. Haverson, University of Bristol, UK), anti-CD172α (DH59B, IgG1, VMRD), anti-CD163 (2A100/11, IgG1, AbD Serotec), anti-MHCII (MSA3, IgG2a, VMRD), anti-TLR2 (1H11, IgG1, provided by Dr. J. Domiguez, Instituto Nacional de Investigación y Tecnología Agraria y Alimentaria, Madrid, Spain), and anti-TLR4 (HTA125, IgG2a, AbD Serotec). As the secondary antibody, AF 647-conjugated mouse isotype-specific goat antisera (Invitrogen) was used. Control samples were stained with secondary antibody only. The threshold for positive cells was set so that control cells stained by secondary antibody only remain ≥ 99% negative (not shown). Flow cytometry was performed using a LSR Fortessa flow cytometer operated by Diva software (Becton Dickinson).

### Bacteria and culture conditions

*Salmonella enterica* serovar Typhimurium 16E5 of porcine origin belonging to phage-type DT104 [41] was used in this study. Bacteria were grown statically in LB broth at 37°C for 18 hours. This culture was diluted 800 × in LB broth and incubated for an additional 6 hours at 37°C to obtain the bacteria in the late logarithmic growth-phase of highly invasive phenotype. Prior to infection of MoMΦ and MoDC, the bacteria were collected by centrifugation and re-suspended in PBS to OD_600_ = 0.3.

### Experimental infection

Prior to infection, the medium was replaced by serum free DMEM without antibiotics. MoMΦ and MoDC were infected with *S.* Typhimurium at a multiplicity of infection equal to 1 for 1 h. Free bacteria were then washed away and gentamicin was added to fresh medium (100 μg/ml) to kill any remaining extracellular bacteria. One hour later, the medium was replaced with fresh medium containing 15 μg/ml gentamicin to prevent multiplication of extracellular bacteria that were eventually released during culture from dead cells. LPS from *S.* Typhimurium (Sigma-Aldrich) at a concentration of 1 μg/ml was used as another stimulus. Negative controls included an assay performed without any contact with *S.* Typhimurium or LPS. Eighteen hours after infection or LPS stimulation, the extracellular transport of proteins was blocked by the addition of 10 μg/ml of Brefeldin A (Sigma-Aldrich). Six hours later, the cells were washed with PBS and lysed by TRI reagent (Sigma-Aldrich) for RNA and protein purification.

### Mass spectrometry and proteome analysis

Proteins from cell cultures were lysed with TRI Reagent and precipitated from the phenolic phase with acetone according to the manufacturer’s recommendations. Dried protein pellets were dissolved in 300 μl of 8 M urea and processed by modified FASP method [42] using a Vivacon 500 device with MWCO of 10 kDa (Sartorius Stedim Biotech). Dissolved proteins were washed twice with 8 M urea and reduced by 10 m M DTT. After reduction proteins were incubated with 50 mM iodoacetamide and washed twice with 25 mM triethylammonium bicarbonate buffer. Trypsin added to sample in 1:50 ratio (w/w) was used as digestive enzyme.

Tryptic peptides were labeled using the stable isotope dimethyl labeling method as described elsewhere [43]. Three combinations of formaldehyde and cyanoborohydride isotopomers were used for labelling; proteins from non-stimulated cells were labeled with a combination of CH_2_O and NaBH_3_CN (light tag), *Salmonella*-infected cells with CD_2_O and NaBH_3_CN (medium tag) and LPS-stimulated cells with ^13^CD_2_O and NaBD_3_CN (heavy tag). Prior to LC-MS/MS analysis, the labeled samples were mixed at a 1:1:1 ratio and analyzed in 3 independent LC-MS/MS runs using the Dionex UltiMate 3000 RSLC nano system connected to a Obitrap Velos Pro mass spectrometer (Thermo Scientific). Such setup enabled semi quantitative analysis of protein abundance. The injected sample was desalted and pre-concentrated during the first 3 min of LC-MS/MS run on the Acclaim PepMap C18 trapping column (2 cm × 75 μm, 3 μm particles) at a flow rate of 5 μl/min using the loading mobile phase consisting of 0.1% formic acid in 98/2 H_2_O/ACN (vol/vol). Chromatographic separation was performed on an EASY-Spray C18 separation column (15 cm × 75 μm, 3 μm particles) at a flow rate of 400 nl/min using the acetonitrile gradient. High resolution (30,000 FWHM at 400 m/z) MS spectra were acquired for the 390-1700 m/z interval. Low resolution MS/MS spectra were acquired in Linear Ion Trap in a data-dependent manner – the top 10 precursors (in terms of abundance) were fragmented using CID fragmentation at a normalized collision energy of 35.

Data were analyzed using the Proteome Discoverer (v.1.4). MS/MS spectra identification was performed by searching SEQUEST against the *Sus scrofa* database (Uniprot, on 4th of September, 2013) and precursor and fragment mass tolerance for searches were 10 ppm and 0.6 Da respectively. Only peptides with a false discovery rate of <1% were included in quantification. Only proteins which quantification was based on 12 or more particular peptide quantifications were considered as reliable. The peptide quantifications were based on ratios of peptide peak areas in stimulated and non-stimulated cells.

### RNA purification, reverse transcription and quantitative real-time PCR

Total RNA was isolated from the water phase of TRI-reagent using RNeasy kit (Qiagen) and immediately reverse transcribed using M-MLV reverse transcriptase (Invitrogen) and oligo-T primer. The resulting cDNA was diluted tenfold is sterile water and used as a template in QRT-PCR immediately or was stored at -20°C until use.

The mRNA sequences related to the LC-MS/MS identified proteins obtained from the GeneBank database were aligned to the genomic sequences using Spidey tool [44] and primers for QRT-PCR were designed over an introns using Primer3 software [45]. The complete list of primer used is available in Additional file 3. QRT-PCR was performed in 3 μl volumes in 384-well-plate format using the QuantiTec SYBR Green PCR Kit (Qiagen) and Innovadyne Nanodrop pipetting station (IDEX Health & Science LLC, Oak Harbor) for PCR mix dispensing. Amplification of PCR products and signal detection were performed using a LightCycler 480 (Roche) with an initial denaturation step at 95°C for 15 min followed by 40 cycles of PCR (95°C for 20 s, 60°C for 30 s and 72°C for 30 s). Each sample was subjected to quantitative real-time PCR in triplicate and only replicates with correct curves and melting temperatures were included in the analysis. The mean values were calculated and used for subsequent analysis.

The expression levels of target genes were determined as follows. The threshold cycle values (C_t_) of genes of interest were normalized against the geometric mean of three the most stable genes identified according to Genorm algorithm [46]. These included hypoxanthine phosphoribosyl transferase I (HPRT), TATA box binding protein 1 (TBP1), and succinate dehydrogenase complex subunit A (SDHA). Tested, but excluded house-keeping genes, included HMBS, ACTB and GAPDH. Finally, the relative expression of each gene of interest was calculated as 2^-ΔCt^.

### Statistical analysis

Data are presented as the mean ± SD or as a fold induction relative to the average expression in non-treated cells. However, for statistical analyses, Ct values obtained by QRT-PCR were used and the statistical significance was determined by one-way ANOVA with Tukey’s post hoc test. (Prism, Graph Pad Software, La Jolla). Differences were considered significant if *p* < 0.05.

## Additional files

Additional file 1:
**The complete list of all proteins identified by LC-MS/MS any time in this study.**
Additional file 2:
**The list of filtered proteins included in QRT-PCR verification.**
Additional file 3:
**List of primers used in this study.**

## Abbreviations

ACN: Acetonitrile
ACTB: Actin Beta
AMCF2: Alveolar Macrophage Chemotactic Factor-2
CDC42: Cell Division Control Protein 42
CID: Collision-Induced Dissociation
CXCL2: Chemokine (C-X-C motif) Ligand 2
DTT: Dithiotreitol
FWHM: Full Width at Half Maximum
GAPDH: Glyceraldehyde 3-phosphate Dehydrogenase
GM-CSF: Granulocyte-Macrophage Colony Stimulating Factor
HMBS: hydroxymethylbilane Synthase
HMOX1: Heme Oxygenase 1
HPRT: Hypoxanthine Phosphoribosyltransferase 1
IL1α: Interleukin-1 Alpha
IL1β: Interleukin-1 Beta
IL4: Interleukin-4
IL8: Interleukin-8
IκBα: Nuclear Factor of Kappa Light Polypeptide Gene Enhancer in B-cells Inhibitor Alpha
ISG15: Interferon Stimulated Gene-15
MARCKSL1: MARCKS-Related Protein
MWCO: Molecular Weight Cut-off
NF-κB: Nuclear Factor of Kappa Light Polypeptide Gene Enhancer in B-cells
OLDLR1: Oxidized Low Density Lipoprotein Receptor 1
OPN: Osteopontin
PAMP: Pathogen Associated Molecular Pattern
PBMC: Peripheral Blood Mononuclear Cells
PPA1: Pyrophosphatase (inorganic) 1
SDHA: Succinate Dehydrogenase Complex Subunit A
SOD2: Superoxid Dismutase
TBP1: TATA Box-Binding Protein
VCAM: Vascular Cell Adhesion Molecule 1

## References

[1] Kampelmacher EH, Edel W, Guinée PA, van Noorle Jansen LM (1969). Experimental salmonella infections in pigs. Zentralblatt Für Veterinärmedizin Reihe B J Vet Med Ser B.

[2] Reed WM, Olander HJ, Thacker HL (1986). Studies on the pathogenesis of salmonella typhimurium and salmonella choleraesuis var kunzendorf infection in weanling pigs. Am J Vet Res.

[3] Schwartz KJ, Straw BE, D’Allaire S, Mengeling WL, Taylor DJ (1999). **Salmonellosis**. Diseases of Swine.

[4] Berends BR, Urlings HA, Snijders JM, Van Knapen F (1996). Identification and quantification of risk factors in animal management and transport regarding salmonella spp. In pigs. Int J Food Microbiol.

[5] Meurens F, Berri M, Auray G, Melo S, Levast B, Virlogeux-Payant I, Chevaleyre C, Gerdts V, Salmon H (2009). Early immune response following salmonella enterica subspecies enterica serovar typhimurium infection in porcine jejunal gut loops. Vet Res.

[6] Collado-Romero M, Arce C, Ramírez-Boo M, Carvajal A, Garrido JJ (2010). Quantitative analysis of the immune response upon salmonella typhimurium infection along the porcine intestinal gut. Vet Res.

[7] Martins RP, Collado-Romero M, Arce C, Lucena C, Carvajal A, Garrido JJ (2013). Exploring the immune response of porcine mesenteric lymph nodes to salmonella enterica serovar typhimurium: an analysis of transcriptional changes, morphological alterations and pathogen burden. Comp Immunol Microbiol Infect Dis.

[8] Rosenberger CM, Finlay BB (2003). Phagocyte sabotage: disruption of macrophage signalling by bacterial pathogens. Nat Rev Mol Cell Biol.

[9] Schnare M, Barton GM, Holt AC, Takeda K, Akira S, Medzhitov R (2001). Toll-like receptors control activation of adaptive immune responses. Nat Immunol.

[10] Carrasco CP, Rigden RC, Schaffner R, Gerber H, Neuhaus V, Inumaru S, Takamatsu H, Bertoni G, McCullough KC, Summerfield A (2001). Porcine dendritic cells generated *in vitro*: morphological, phenotypic and functional properties. Immunology.

[11] Auray G, Facci MR, van Kessel J, Buchanan R, Babiuk LA, Gerdts V (2010). Differential activation and maturation of two porcine DC populations following TLR ligand stimulation. Mol Immunol.

[12] Facci MR, Auray G, Buchanan R, van Kessel J, Thompson DR, Mackenzie-Dyck S, Babiuk LA, Gerdts V (2010). A comparison between isolated blood dendritic cells and monocyte-derived dendritic cells in pigs. Immunology.

[13] Raymond CR, Wilkie BN (2005). Toll-like receptor, MHC II, B7 and cytokine expression by porcine monocytes and monocyte-derived dendritic cells in response to microbial pathogen-associated molecular patterns. Vet Immunol Immunopathol.

[14] Sun SC, Ganchi PA, Ballard DW, Greene WC (1993). NF-kappa B controls expression of inhibitor I kappa B alpha: evidence for an inducible autoregulatory pathway. Science.

[15] Kapetanovic R, Fairbairn L, Beraldi D, Sester DP, Archibald AL, Tuggle CK, Hume DA (2012). Pig bone marrow-derived macrophages resemble human macrophages in their response to bacterial lipopolysaccharide. J Immunol Baltim Md 1950.

[16] Guzylack-Piriou L, Alves MP, McCullough KC, Summerfield A (2010). Porcine Flt3 ligand and its receptor: generation of dendritic cells and identification of a new marker for porcine dendritic cells. Dev Comp Immunol.

[17] Islam MA, Pröll M, Hölker M, Tholen E, Tesfaye D, Looft C, Schellander K, Cinar MU (2013). Alveolar macrophage phagocytic activity is enhanced with LPS priming, and combined stimulation of LPS and lipoteichoic acid synergistically induce pro-inflammatory cytokines in pigs. Innate Immun.

[18] Minakawa M, Sone T, Takeuchi T, Yokosawa H (2008). Regulation of the nuclear factor (NF)-kappaB pathway by ISGylation. Biol Pharm Bull.

[19] Hiscott J, Marois J, Garoufalis J, D’Addario M, Roulston A, Kwan I, Pepin N, Lacoste J, Nguyen H, Bensi G (1993). Characterization of a functional NF-kappa B site in the human interleukin 1 beta promoter: evidence for a positive autoregulatory loop. Mol Cell Biol.

[20] Mori N, Prager D (1996). Transactivation of the interleukin-1alpha promoter by human T-cell leukemia virus type I and type II Tax proteins. Blood.

[21] Keates AC, Keates S, Kwon JH, Arseneau KO, Law DJ, Bai L, Merchant JL, Wang TC, Kelly CP (2001). ZBP-89, Sp1, and nuclear factor-kappa B regulate epithelial neutrophil-activating peptide-78 gene expression in Caco-2 human colonic epithelial cells. J Biol Chem.

[22] Kunsch C, Rosen CA (1993). NF-kappa B subunit-specific regulation of the interleukin-8 promoter. Mol Cell Biol.

[23] Xu Y, Kiningham KK, Devalaraja MN, Yeh CC, Majima H, Kasarskis EJ, St Clair DK (1999). An intronic NF-kappaB element is essential for induction of the human manganese superoxide dismutase gene by tumor necrosis factor-alpha and interleukin-1beta. DNA Cell Biol.

[24] Schumann RR (1995). Mechanisms of transcriptional activation of lipopolysaccharide binding protein (LBP). Prog Clin Biol Res.

[25] Klaman LD, Thorley-Lawson DA (1995). Characterization of the CD48 gene demonstrates a positive element that is specific to epstein-barr virus-immortalized B-cell lines and contains an essential NF-kappa B site. J Virol.

[26] Samant RS, Clark DW, Fillmore RA, Cicek M, Metge BJ, Chandramouli KH, Chambers AF, Casey G, Welch DR, Shevde LA (2007). Breast cancer metastasis suppressor 1 (BRMS1) inhibits osteopontin transcription by abrogating NF-kappaB activation. Mol Cancer.

[27] Nagase M, Abe J, Takahashi K, Ando J, Hirose S, Fujita T (1998). Genomic organization and regulation of expression of the lectin-like oxidized low-density lipoprotein receptor (LOX-1) gene. J Biol Chem.

[28] Lavrovsky Y, Schwartzman ML, Levere RD, Kappas A, Abraham NG (1994). Identification of binding sites for transcription factors NF-kappa B and AP-2 in the promoter region of the human heme oxygenase 1 gene. Proc Natl Acad Sci U S A.

[29] Aderem A (1992). The role of myristoylated protein kinase C substrates in intracellular signaling pathways in macrophages. Curr Top Microbiol Immunol.

[30] Blackshear PJ (1993). The MARCKS family of cellular protein kinase C substrates. J Biol Chem.

[31] Yue L, Lu S, Garces J, Jin T, Li J (2000). Protein kinase C-regulated dynamitin-macrophage-enriched myristoylated alanine-rice C kinase substrate interaction is involved in macrophage cell spreading. J Biol Chem.

[32] Wu M, Chen DF, Sasaoka T, Tonegawa S (1996). Neural tube defects and abnormal brain development in F52-deficient mice. Proc Natl Acad Sci U S A.

[33] Chen J, Chang S, Duncan SA, Okano HJ, Fishell G, Aderem A (1996). Disruption of the MacMARCKS gene prevents cranial neural tube closure and results in anencephaly. Proc Natl Acad Sci U S A.

[34] Seykora JT, Ravetch JV, Aderem A (1991). Cloning and molecular characterization of the murine macrophage “68-kDa” protein kinase C substrate and its regulation by bacterial lipopolysaccharide. Proc Natl Acad Sci U S A.

[35] Sunohara JR, Ridgway ND, Cook HW, Byers DM (2001). Regulation of MARCKS and MARCKS-related protein expression in BV-2 microglial cells in response to lipopolysaccharide. J Neurochem.

[36] Rosé SD, Byers DM, Morash SC, Fedoroff S, Cook HW (1996). Lipopolysaccharide stimulates differential expression of myristoylated protein kinase C substrates in murine microglia. J Neurosci Res.

[37] Chang S, Stacey KJ, Chen J, Costelloe EO, Aderem A, Hume DA (1999). Mechanisms of regulation of the MacMARCKS gene in macrophages by bacterial lipopolysaccharide. J Leukoc Biol.

[38] Mancek-Keber M, Bencina M, Japelj B, Panter G, Andrä J, Brandenburg K, Triantafilou M, Triantafilou K, Jerala R (2012). MARCKS as a negative regulator of lipopolysaccharide signaling. J Immunol Baltim Md 1950.

[39] Wang X, Eaton M, Mayer M, Li H, He D, Nelson E, Christopher-Hennings J (2007). Porcine reproductive and respiratory syndrome virus productively infects monocyte-derived dendritic cells and compromises their antigen-presenting ability. Arch Virol.

[40] Stepanova H, Pavlova B, Stromerova N, Ondrackova P, Stejskal K, Slana I, Zdrahal Z, Pavlik I, Faldyna M (2012). Different immune response of pigs to Mycobacterium avium subsp. avium and Mycobacterium avium subsp. hominissuis infection. Vet Microbiol.

[41] Pavlova B, Volf J, Ondrackova P, Matiasovic J, Stepanova H, Crhanova M, Karasova D, Faldyna M, Rychlik I (2011). SPI-1-encoded type III secretion system of Salmonella enterica is required for the suppression of porcine alveolar macrophage cytokine expression. Vet Res.

[42] Wiśniewski JR, Zougman A, Nagaraj N, Mann M (2009). Universal sample preparation method for proteome analysis. Nat Methods.

[43] Boersema PJ, Raijmakers R, Lemeer S, Mohammed S, Heck AJR (2009). Multiplex peptide stable isotope dimethyl labeling for quantitative proteomics. Nat Protoc.

[44] Wheelan SJ, Church DM, Ostell JM (2001). Spidey: a tool for mRNA-to-genomic alignments. Genome Res.

[45] Koressaar T, Remm M (2007). Enhancements and modifications of primer design program Primer3. Bioinforma Oxf Engl.

[46] Vandesompele J, De Preter K, Pattyn F, Poppe B, Van Roy N, De Paepe A, Speleman F (2002). Accurate normalization of real-time quantitative RT-PCR data by geometric averaging of multiple internal control genes. Genome Biol.

