# Supplementary material for: The response of porcine monocyte derived macrophages and dendritic cells to Salmonella Typhimurium and lipopolysaccharide
Source: BMC Vet Res. 2014 Oct 1;10:244. doi: 10.1186/s12917-014-0244-1 (PMC4195948; doi:10.1186/s12917-014-0244-1)
Supplement: Additional file 3: — List of primers used in this study. [file 12917_2014_244_MOESM3_ESM.doc]

Additional file 3

List of primers used

| **Gene** | **Gene abbreviation** | **Forward primer** | **Reverse primer** | **Acc. No.;** | **Product length** | **Refference** |
| --- | --- | --- | --- | --- | --- | --- |
| Oxidized low density lipoprotein receptor 1 | OLR1 | gatgcccaactgctgaagat | gcagctccctggagtctaaa | NM_213805 | 176 | this study |
| Pyrophosphatase 1 (anorganic) | PPA1 | aataccaccgtgtccgagag | tttttctggtgatggaaccac | XM_001925080 | 137 | this study |
| AMCF2 (porcine homolog of CXCL5/6) | AMCF2 | gagaaaggatcatgcattgga | tcccacccctctctcctagt | NM_213876 | 152 | this study |
| CD48 | CD48 | ccaagtcagcaatcctgtga | aggaggctaagaacggtggt | NM_001243714 | 135 | this study |
| MARCKS-related protein | MARCKSL1 | gacgtgaccaccgaggag | tgggggataagtctccattg | XM_003127758 | 124 | this study |
| NF-κB inhibitor alpha | IκBα | acgagcagatggtgaaggag | tcatggatgatggccaagt | NM_001005150 | 130 | [1] |
| Annexin A1 | ANXA1 | ctggacctggagttgaaagg | aatttcagaacgggaaacca | NM_001163998 | 165 | this study |
| Vascular cell adhesion molecule 1 | VCAM1 | atccaagctgctccaaaaga | ggccctgtggatggtatatg | NM_213891 | 189 | this study |
| Superoxide dismutase 2, mitochondrial | SOD2 | gctcggtttcaacaaggaac | taatacgcatgctcccacac | NM_214127 | 123 | this study |
| Interleukin-1 beta | IL1β | gggacttgaagagagaagtgg | ctttcccttgatccctaaggt | NM_214055 | 95 | [3] |
| Interleukin-8 | IL8 | ttctgcagctctctgtgaggc | ggtggaaaggtgtggaatgc | NM_213867 | 92 | [1] |
| Heme oxygenase 1 | HMOX1 | ccttagtgtcctgggtcagc | gtcctcgaagaagccaagac | NM_001004027 | 86 | this study |
| ISG15 ubiquitin-like modifier | ISG15 | gatgctgggaggcaagga | caggatgctcagtgggtctct | NM_001128469 | 229 | this study |
| Chemokine (C-X-C motif) ligand 2 | CXCL2 | ggaagtttgtctcaaccccgcagc | tctacatcagttggcactgctcttgtttagc | NM_001001861 | 85 | this study |
| Interleukin 1-alpha | IL1α | tgtgaagtgttgacaggccgtatgtacc | ctcagcacatgctcagcgagtgac | NM_214029 | 85 | this study |
| Cell division cycle 42 GTP binding protein | CDC42 | ccagagactgctgaaaagctg | cagccaatattgcttcgtca | XM_005656039 | 112 | this study |
| Prefoldin subunit 2 | PFDN2 | gaaaatgctaccgcatggtt | ttcttttccctttgcctgaa | XM_001927323 | 142 | this study |
| Nuclear factor of kappa light polypeptide gene | NF-κB1 | ccctgtgaagaccacctctc | aagctgagtttgcgaaagga | NM_001048232 | 132 | this study |
| Proteasome (macropain) subunit, beta type, 1 | PSMB1 | tgtggggtcctaccagagag | tgctccacattttgcatgtt | NM_001244353 | 111 | this study |
| Proteasome (macropain) subunit, beta type, 4 | PSMB4 | tgcagagaccaactgggata | atctctgaaccagccacgtt | NM_001244455 | 118 | this study |
| Serglycin | SRGN | cctccaaggactgacccttt | catttagggagccacttcca | XM_005657391 | 121 | this study |
| Osteopontin | OPN | tgagctggtcaccgatttcc | ggtcttcctctgtggcatcc | NM_214023 | 173 | this study |
| Hypoxanthin phosphoribosyl transferase | HPRT | cggctccgttatggcg | ggtcataacctggttcgtcatca | NM_001032376 | 91 | [2] |
| TATA box binding protein 1 | TBP1 | aacagttcagtagttatgagccaga | agatgttctcaaacgcttcg | DQ845178 | 153 | [4] |
| Succinate dehydrogenase complex subunit A | SDHA | ctacaaggggcaggttctga | aagacaacgaggtccaggag | DQ845177 | 141 | [4] |

1. Kyrova K, Stepanova H, Rychlik I, Faldyna M, Volf J: **SPI-1 encoded genes of Salmonella Typhimurium influence differential polarization of porcine alveolar macrophages in vitro**. *BMC Vet Res* 2012, **8**:115.

2. Volf J, Boyen F, Faldyna M, Pavlova B, Navratilova J, Rychlik I: **Cytokine response of porcine cell lines to Salmonella enterica serovar typhimurium and its hilA and ssrA mutants**. *Zoonoses Public Health* 2007, **54**:286–293.

3. Von der Hardt K, Kandler MA, Fink L, Schoof E, Dötsch J, Brandenstein O, Bohle RM, Rascher W: **High frequency oscillatory ventilation suppresses inflammatory response in lung tissue and microdissected alveolar macrophages in surfactant depleted piglets**. *Pediatr Res* 2004, **55**:339–346.

4. Nygard A-B, Jørgensen CB, Cirera S, Fredholm M: **Selection of reference genes for gene expression studies in pig tissues using SYBR green qPCR**. *BMC Mol Biol* 2007, **8**:67.
